# Supracricoid partial laryngectomy and reconstruction of the anterior epiglottic space flap: a new surgical approach for supracricoid partial laryngectomy

**DOI:** 10.3389/fonc.2025.1521929

**Published:** 2025-05-29

**Authors:** Chenggang Mao, Zhiqun He, Linglong Liu, Yi Zhang, Fei Chen, Xi Liang

**Affiliations:** ^1^ Department of Otolaryngology Head and Neck Surgery, Jingzhou Hospital Affiliated to Yangtze University, Jingzhou, China; ^2^ Department of Otolaryngology, Head and Neck Surgery, West China Hospital, Sichuan University, Chengdu, China

**Keywords:** laryngeal tumor, supracricoid partial laryngectomy, reconstruction surgery, ESFR, CHEP results follow-up (44-116 months, 94.7% rate) revealed similar 3-year (CHEP: 90.6%, ESFR: 91.5%, P>0.05) and 5-year (CHEP: 83.3%)

## Abstract

**Objective:**

To introduce a novel surgical technique for partial laryngectomy involving the reconstruction of the epiglottic space flap (ESFR) on the cricoid cartilage, and to compare its clinical efficacy and functional outcomes with those of cricohyoidoepiglottopexy (CHEP) in the treatment of laryngeal squamous cell carcinoma, exploring the feasibility and clinical significance of this new surgical approach.

**Methods:**

A retrospective analysis was conducted on 57 patients with laryngeal squamous cell carcinoma who were treated between January 2014 and January 2020. The inclusion criteria were suitability for CHEP according to the 2002 UICC criteria and the absence of anterior epiglottic space invasion. Postoperative complications, glottic area status, recurrence, and survival were compared between the CHEP group (n=22) and the ESFR group (n=35).

**Results:**

Follow-up (44-116 months; 94.7% rate) revealed similar 3-year (CHEP: 90.6%; ESFR: 91.5%; P>0.05) and 5-year (CHEP: 83.3%; ESFR: 89.3%; P>0.05) cumulative survival. ESFR significantly reduced extubation time (ESFR: 8 ± 2.5 days; CHEP: 18 ± 3.1 days; P<0.01) and swallowing errors (ESFR: 5.7%; CHEP: 22.7%; P<0.05). No significant differences were observed in pharyngeal fistula, laryngeal stenosis, or recurrence rates (P>0.05).

**Conclusion:**

Compared to CHEP, ESFR technique demonstrates equivalent surgical eligibility criteria and oncological resection margins. However, ESFR uniquely preserves the anatomical integrity of the laryngeal framework, enabling superior postoperative functional outcomes through expedited restoration of phonatory and deglutitive capacities while maintaining long-term laryngeal preservation.

## 1 Introduction

In recent years, the treatment strategy for laryngeal cancer has gradually shifted from total laryngectomy to function-preserving surgeries (such as supracricoid partial laryngectomy, SCPL) and non-surgical treatments (such as radiotherapy). Multiple studies (1–3) have shown that for early-stage laryngeal cancer, the survival rates of radiotherapy and surgery are similar, but function-preserving surgeries have advantages in local control and long-term quality of life. The landmark RTOG 91-11 trial (2) showed that for advanced laryngeal cancer, chemoradiotherapy achieves laryngeal preservation rates similar to total laryngectomy but has higher long-term dysphagia and gastrostomy dependence rates in the chemoradiotherapy group, highlighting the trade-offs between organ preservation and functional morbidity. In contrast, function-preserving surgeries provide robust local control while maintaining laryngeal integrity, especially for T3-T4a lesions where surgical margins can be achieved.

Supracricoid partial laryngectomy (SCPL) is a widely accepted surgical technique applicable to both glottic and supraglottic laryngeal cancers, as well as cases of radiotherapy failure and postoperative laryngeal stenosis. SCPL encompasses two main procedures: cricohyoidoepiglottopexy (CHEP) and cricohyoidopexy (CHP) (4). While SCPL offers advantages such as a relatively simple surgical procedure and good local control rates, it is associated with a higher incidence of postoperative dysphagia and aspiration, along with prolonged extubation times, leading to patient discomfort (5). The emergence of novel approaches, epiglottic space flap reconstruction (ESFR), addresses these limitations by preserving laryngeal framework integrity and minimizing neurovascular disruption. In patients eligible for ESFR, the epiglottic space can be dissected and inferiorly rotated to create a tissue flap for laryngeal reconstruction. This technique, known as supracricoid partial laryngectomy with ESFR, maintains the normal anatomical position of the larynx. Clinical experience indicates that ESFR significantly reduces the incidence of postoperative dysphagia and aspiration, and shortens extubation time. Since January 2016, our institution has adopted ESFR with satisfactory clinical outcomes. This report presents the clinical data of 35 patients with laryngeal cancer who underwent ESFR, comparing and analyzing them with 22 patients who underwent CHEP.

## 2 Materials and methods

### 2.1 Research object

A retrospective analysis was conducted on 57 patients with laryngeal cancer admitted to the Department of Otolaryngology, Head and Neck Surgery at West China Hospital of Sichuan University from January 2016 to January 2022. This study utilized the hospital’s electronic medical records (EMR). Patients were identified through a structured search of surgical codes (e.g., ICD-10 codes for laryngeal cancer: C32.x) and procedural terms (e.g., “CHEP,” “ESFR,” “partial laryngectomy”) within the EMR system.

#### 2.1.1 Inclusion criteria

- Pathologically confirmed laryngeal squamous cell carcinoma;
- Tumor not invading the pre-epiglottic space;
- Meeting the CHEP criteria according to the 2002 UICC standard.

#### 2.1.2 Exclusion criteria

- Distant metastasis (M1);
- Bilateral fixation of the arytenoid cartilages;
- Invasion of the perichondrium of the thyroid cartilage.

The CHEP group comprised 22 patients (21 males, 1 female), with ages ranging from 45 to 73 years (median age: 57 years). Clinical staging was based on the 2002 UICC TNM staging criteria. Among these patients, there were 22 cases of glottic laryngeal cancer, including 2 cases classified as T2N0M0, 6 as T2N1M0, 9 as T3N0M0, and 5 as T3N1M0. The ESFR group included 35 patients (33 males, 2 females), with ages ranging from 46 to 75 years (median age: 61 years). All 35 patients in this group had glottic carcinoma, with the following staging distribution: 5 cases of T2N0M0, 7 cases of T2N1M0, 12 cases of T3N0M0, 10 cases of T3N1M0, and 1 case of T4N1M0.

### 2.2 Surgical procedure

#### 2.2.1 CHEP surgery

General anesthesia was induced via endotracheal intubation. An arc-shaped incision was made in the anterior neck region, followed by the separation of the anterior cervical muscles. The sternohyoid and thyrohyoid muscles were horizontally transected at the upper edge of the thyroid cartilage, and the sternohyoid muscle along with the bilateral pharyngeal constrictor muscles were subsequently cut. The cricothyroid membrane was horizontally incised, the thyrohyoid membrane was excised, and the laryngeal cavity was entered from a superior approach. Starting from the less affected side of the lesion, an incision was made in front of the arytenoid cartilage, taking care to preserve the vocal process and the cricoarytenoid muscle group. The thyroid cartilage was then split along its midline, allowing the laryngeal cavity to be opened in a book-like fashion, and the severely affected contralateral side was removed. Three absorbable sutures (size 1) were passed through the cricoid cartilage under the mucosa, then through the remaining epiglottic cartilage and the anterior epiglottic space, bypassing the hyoid bone to reach the tongue base muscle, and cricoid cartilage hyoid epiglottic fixation was performed. The pharyngeal cavity was closed, and the incision was reinforced by suturing the anterior cervical muscle layer. A tracheotomy was performed at the lowest point of the curved incision in the anterior neck.

#### 2.2.2 ESFR surgery

**Figure 1:**
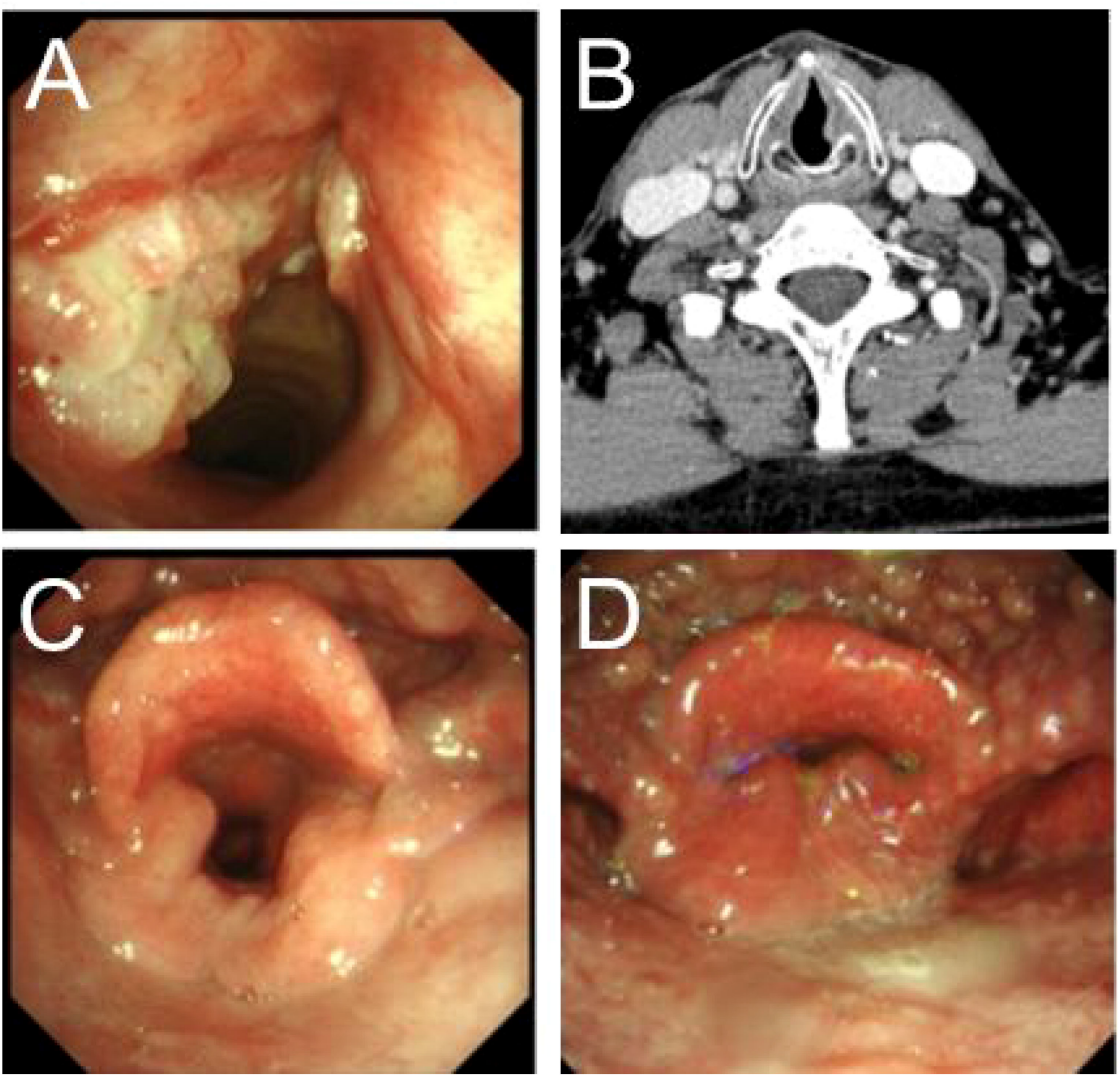
Presurgery and postsurgery glottis of patients of supracricoid partial laryngectomy with ESFR surgery. **(A)** The larynx lesion was examined by preoperative laryngoscopy. **(B)** Preoperative enhanced CT for laryngeal lesions. **(C)** Laryngoscopy the opened glottis six months after surgery. **(D)** Laryngoscopy the closed glottis six months after surgery.

**Figure 2:**
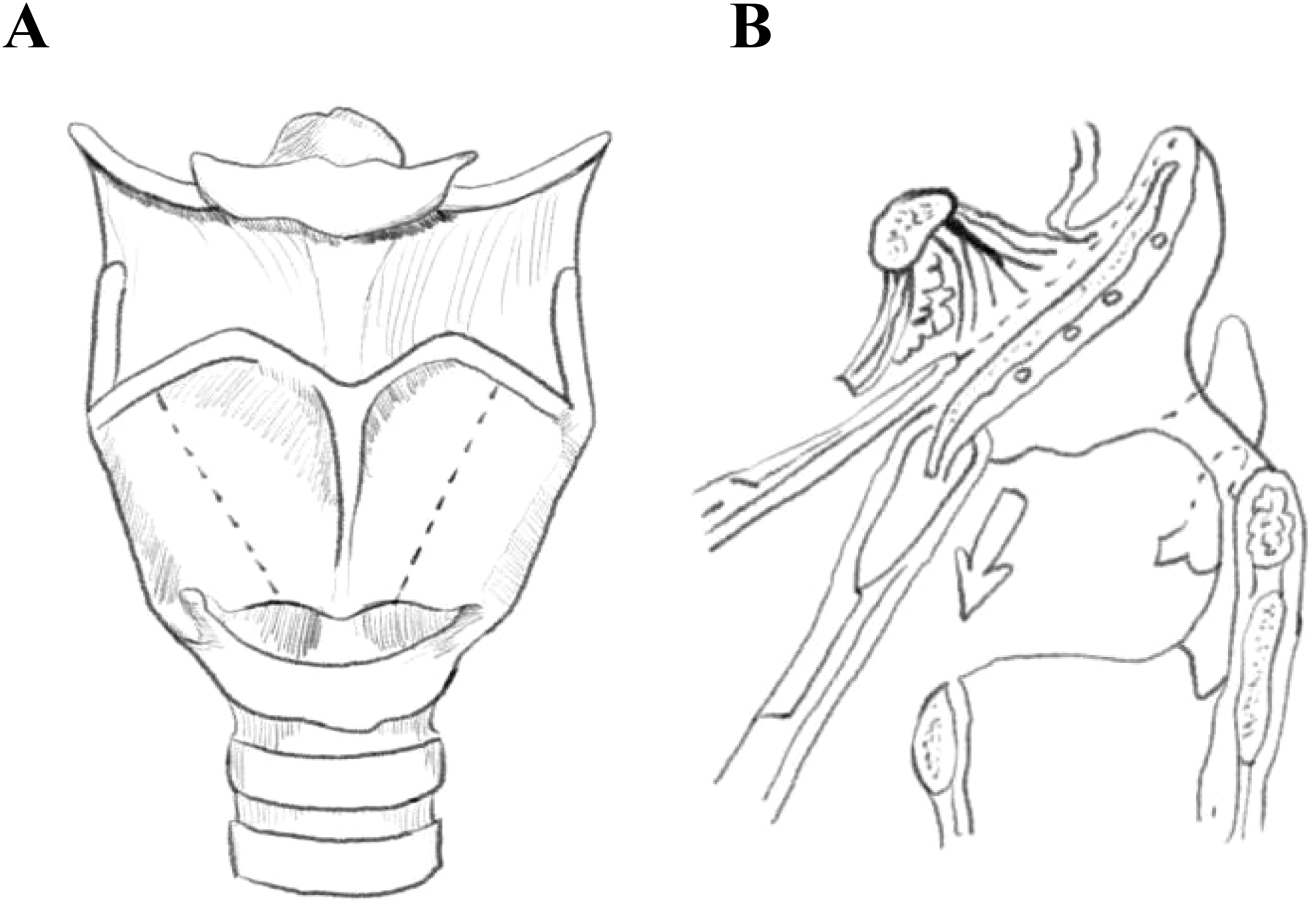
Schematic drawing of supracricoid partial laryngectomy with epiglottic space flap reconstruction(ESFR) surgery. **(A)** The dotted line depicts the region of thyroid cartilage resected during ESFR. **(B)** The dotted line delineates the intralaryngeal region resected during ESFR.

**Figure 3:**
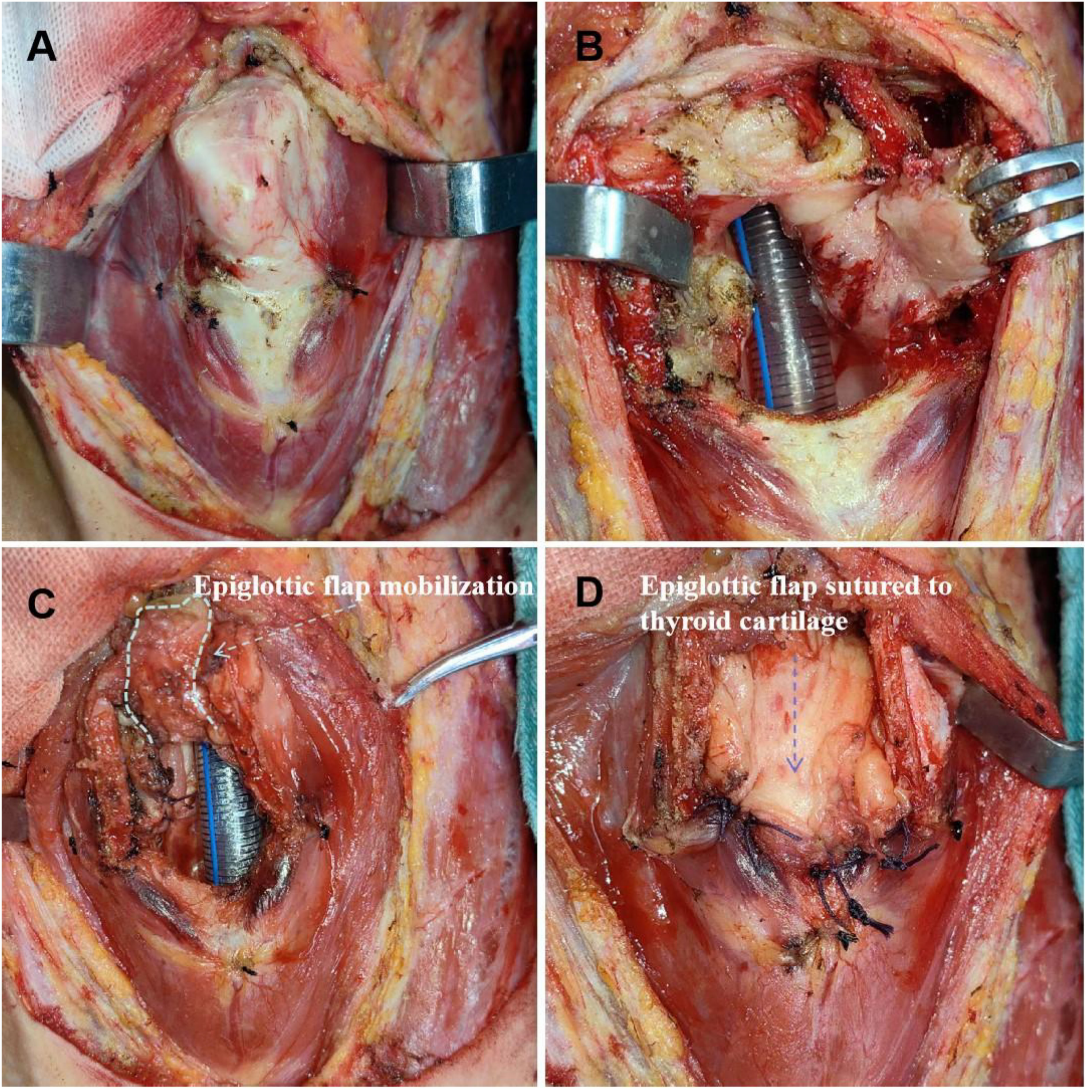
Supracricoid partial laryngectomy with ESFR surgical procedure. **(A)** The thyroid cartilage was cut about 1/3 to 1/2 away from the mediolateral cartilage and removed. **(B)** The laryngeal cavity was exposed and the lesion was removed. **(C)** Hold the root of the epiglottis, free the preepiglottic space, release the downward epiglottis. Epiglottic flap mobilization (white dashed line) **(D)** The epiglottis space flap was closed with the surrounding thyroid cartilage and closed to form a new laryngeal cavity (blue dashed line).

With the patient under general anesthesia and an ascending intubation in place, a curved incision is made in the anterior neck region. The anterior cervical muscles are then separated in the midline, and a thyroid hook is used to retract these muscles laterally, thereby fully exposing the thyroid cartilage. The extent of thyroid cartilage resection in ESFR surgery is carefully determined based on tumor location, identified through preoperative visual inspection and image-guided evaluations, including CT (**Figure 1A, B**). Intraoperative frozen section pathology is used to ensure adequate oncological resection margins, confirmed postoperatively by histopathology. This approach allows for optimal removal of malignant tissue while preserving sufficient thyroid cartilage for functional reconstruction. The resection typically involves one-third to one-half of the thyroid cartilage along its lateral and upper aspects from both sides, which differs from classical frontolateral partial laryngectomies. Use an electric knife to cut the thyroid cartilage membrane along the upper edge of the bilateral thyroid cartilage plates, and make an arc-shaped incision until it converges at the cricoid thyroid membrane (**Figure 2A, B**). One-third to one-half of the thyroid cartilage is resected along its lateral and upper aspects from both sides, which differs from classical frontolateral partial laryngectomies (**Figure 3A**). The thyroid cartilage is incised along the electric knife’s cut line to expose the laryngeal cavity, where the tumor is removed, ensuring surgical safety (**Figure 3B**). The epiglottis root is clamped, and the fibrous adipose tissue, hyoid epiglottic ligament, lingual epiglottic ligament, and epiglottic lingual mucosa in the anterior epiglottic space are freed from bottom to top. The epiglottis is then released and moved downward, with care taken to maintain the integrity of the mucosa attached to the free edge of the epiglottis to ensure blood supply to the epiglottic valve (**Figure 3C**). The epiglottic valve is pulled down. Three absorbable sutures (size 1) are used to close the glottis flap and cricoid cartilage, sealing the laryngeal cavity. The edge of the epiglottic valve is sutured to the residual thyroid cartilage tissue and the base of the tongue to further seal the laryngeal cavity (**Figure 3D**). The central part of the epiglottic valve is sutured to the thyroid cartilage to expand the pharyngeal cavity. A drainage tube is placed, the anterior cervical muscle layer is reinforced, the incision is sutured, and a tracheotomy is performed.

### 2.3 Postoperative outcome analyses

Patients were followed up to evaluate 3-year and 5-year cumulative survival rates. Extubation time and extubation rates were compared between the two groups. The incidence and recurrence rates of complications, including pharyngeal fistula and laryngeal stenosis, were also assessed.

### 2.4 Statistical analyses

The statistical analysis of survival data for patients with laryngeal cancer was conducted using SPSS 23.0 software. The 3-year and 5-year survival rates were analyzed using the Kaplan-Meier method, and the differences in survival rates between different surgical procedures were compared using the log-rank test. Multivariate Cox proportional hazards regression was performed to adjust for potential confounders, including age (continuous variable), T-stage (T2 vs. T3), and nodal status (N0 vs. N1). For the comparison of postoperative extubation rates between two groups, the four grid exact test method was used. The comparison of postoperative extubation time between the two groups was conducted using the t-test. To further mitigate selection bias, a *post hoc* propensity score-matched analysis (1:1 matching with caliper = 0.2) was conducted using SPSS 23.0. Matching variables included age (± 5 years), sex, T-stage, and nodal status. Standardized mean differences (SMD) were calculated to assess balance between groups after matching (SMD <0.1 indicated negligible imbalance).

### 2.5 Minimizing selection bias

To mitigate potential selection bias, the following measures were implemented:

1. Consecutive enrollment: all patients meeting inclusion criteria during the study periods were consecutively enrolled to avoid selection of favorable cases.
2. Propensity score matching: groups were balanced for age, sex, and T/N-stage using a 1:1 matching algorithm.
3. Blinded outcome assessment: postoperative complications (e.g., dysphagia, laryngeal stenosis) and survival outcomes were evaluated by clinicians uninvolved in surgical procedures.
4. Multivariate adjustments: Cox regression models adjusted for age, T-stage, and nodal status to control for residual confounding factors.

## 3 Results

### 3.1 Survival rates

Both the CHEP group (n=22) and the ESFR group (n=35) demonstrated favorable postoperative survival rates. Follow-up data for the CHEP group revealed that of the 22 patients, all had completed at least 3 years of follow-up, with 2 deaths and 1 loss to follow-up reported. Of the CHEP group, 19 patients had completed 5 years of follow-up, with 3 deaths and 1 loss to follow-up. In the ESFR group, all 35 patients completed at least 3 years of follow-up, with 1 death and 2 losses to follow-up. Of these, 32 patients completed 5 years of follow-up, with 3 deaths and 1 loss to follow-up.

**Figure 4:**
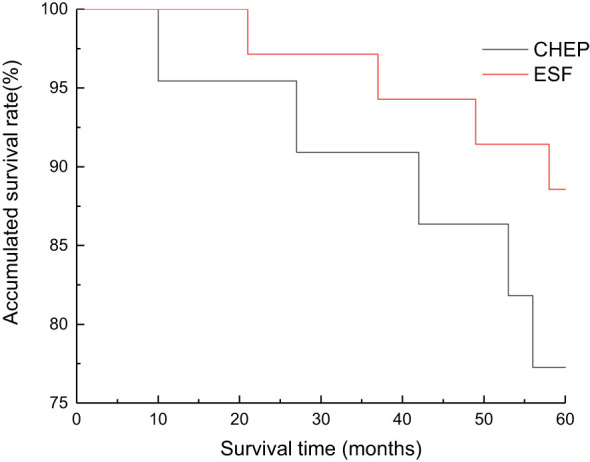
Overall survival curves of supracricoid partial laryngectomy with ESFR group and CHEP group. There was no significant difference in 3 and 5 year cumulative survival between ESFR and CHEP groups.

The Kaplan-Meier analysis of the original cohort (n=57) demonstrated comparable 3-year and 5-year survival rates between the CHEP and ESFR groups. Specifically, the 3-year survival rates were 90.6% (95% CI: 85.2–95.0) for the CHEP group and 91.5% (95% CI: 86.7–96.3) for the ESFR group (*P*=0.925). The 5-year survival rates were 83.3% for the CHEP group and 89.3% for the ESFR group (*P*=0.873) (**Figure 4**). After propensity score matching (n=40, 20 pairs), the survival outcomes remained consistent. The 3-year survival rates were 89.5% (95% CI: 84.1–94.9%) for the CHEP group and 90.0% (95% CI: 85.0–95.0%) for the ESFR group (*P*=0.91). The 5-year survival rates were 82.4% (95% CI: 75.3–89.5%) for the CHEP group and 87.5% (95% CI: 80.8–94.2%) for the ESFR group (*P*=0.72). Multivariate Cox regression analysis confirmed no significant survival difference between the two groups, with a hazard ratio of 1.12 (95% CI: 0.78–1.61, *P*=0.54), even after adjusting for age and T-staging.

### 3.2 Extubation time and rate

The postoperative extubation time was 18 ± 3.1 days in the CHEP group and 8 ± 2.5 days in the ESFR group, showing a statistically significant difference (t=3.50, P<0.01). The postoperative extubation rate was 95.5% (21/22) in the CHEP group and 100% (35/35) in the ESFR group, with no statistically significant difference (P>0.05). After 8 weeks of postoperative evaluation, the incidence of swallowing errors was 22.7% (5/22) in the CHEP group and 5.7% (2/35) in the ESFR group. The difference between the two groups was statistically significant (P<0.05) as determined by the precision test method.

### 3.3 Postoperative complications

Both surgical procedures had manageable complications. Pharyngeal fistulas were treated with antiseptic dressings and prophylactic antibiotics, healing within 10-14 days without surgical revision. Patients with fistulas had a median hospital stay of 14 days, compared to 12 days for the CHEP group and 9 days for the ESFR group without complications. No laryngeal stenosis cases were observed, attributed to preserved thyroid cartilage scaffolding in ESFR and early vocal rehabilitation. Standardized protocols, including multidisciplinary reviews and serial videofluoroscopic swallowing assessments, guided dietary progression and complication mitigation, minimizing reintervention needs and optimizing recovery.

Postoperative pharyngeal fistula occurred in one patient (4.5%) in the CHEP group and one patient (2.9%) in the ESFR group; both cases resolved with wound care. Postoperative recurrence was observed in three patients (13.6%) in the CHEP group and four patients (11.4%) in the ESFR group. No instances of laryngeal stenosis were observed in either group during the follow-up period.

## 4 Discussion

Recent retrospective analyses, including a 2023 Frontiers study (6), have reinforced the role of surgery in select populations. This multicenter retrospective series of 1,452 patients with T2-T3 laryngeal squamous cell carcinoma reported superior 5-year disease-specific survival rates for surgical cohorts (82% vs. 68% for chemoradiotherapy, P<0.01), particularly in tumors with paraglottic or subglottic extension. Compared to radiotherapy, the surgical treatment preserves laryngeal function through anatomical reconstruction and avoids radiation-related complications such as chondronecrosis. For T3-T4a tumors, surgical local control rates may be superior to those achieved with chemoradiotherapy (7).

The fundamental principle of laryngeal cancer surgery involves achieving complete tumor excision while optimizing patient survival, with concurrent prioritization of laryngeal function preservation and postoperative quality of life. The CHEP procedure, originally developed in Europe, represents a significant advancement in functional laryngeal surgery. This technique not only ensures oncological radicality for advanced laryngeal carcinomas but also demonstrates comparable survival outcomes to total laryngectomy while substantially improving quality of life metrics (8). Our institution has undertaken systematic investigation of this surgical approach in recent years. The CHEP cohort exhibited 3-year and 5-year cumulative survival rates of 90.6% and 83.3% respectively, while the ESFR group demonstrated corresponding rates of 91.5% and 89.3%. Consistent with previous reports (9), no statistically significant difference in 3- or 5-year cumulative survival was observed between the two groups.

The rising incidence of laryngeal cancer has spurred the development of varied surgical techniques, with partial laryngectomy representing a substantial proportion (20%-79%) of all laryngeal cancer surgeries (10, 11). Capitalizing on the unique embryological development, anatomical structure, and lymphatic drainage of the larynx, advancements in surgical methods have facilitated the wider adoption of laryngeal function-sparing procedures. These approaches not only improve quality of life but also yield favorable long-term survival outcomes (12). Our department’s experience in laryngeal cancer management has evolved from total laryngectomy to partial laryngectomy, incorporating techniques such as CHEP, CHP, and various innovative reconstructive approaches tailored for partial laryngectomy. While these methods preserve laryngeal function and achieve good survival rates, we observed a higher incidence of postoperative dysphagia and prolonged extubation times with the traditional CHEP procedure. Consequently, since 2014, we have refined the original CHEP technique and adopted the ESFR surgical method, achieving improved clinical results. ESFR preserves laryngeal function by retaining portions of the thyroid cartilage lamina and the epiglottic flap, which maintains the anatomical height of the laryngeal cavity and the configuration of the pyriform sinus, thereby reducing postoperative aspiration (**Figure 1C, D**). Furthermore, because the cricothyroid joint does not require division, the risk of recurrent laryngeal nerve injury is diminished, promoting early recovery of laryngeal function (13).

At present, both CHEP and ESFR are frequently employed surgical techniques at West China Hospital of Sichuan University. ESFR represents an advancement over CHEP, significantly reducing the incidence of swallowing errors. Given ESFR’s capacity to preserve a substantial portion of the thyroid cartilage plates contingent upon the lesion’s condition, this study proposes the following optimal indications: ① T2 glottic carcinoma characterized by limited vocal cord mobility and invasion of the laryngeal ventricle; glottic laryngeal cancer with subglottic invasion not exceeding 1 cm. ② T3 glottic carcinoma with invasion of the paraglottic space. Particularly for patients whose tumors involve the contralateral vocal cords but who retain normal function of the contralateral cricoarytenoid joint. In addition to the aforementioned scenarios, traditional CHEP surgery remains a viable option for T4 glottic carcinoma, even when the thyroid cartilage is locally invaded, provided that the outer periosteum remains intact.The selection criteria for ESFR are more stringent than those for traditional CHEP, which has contraindications including glottic tumors with bilateral arytenoid cartilage fixation or invasion of the arytenoid region, extensive subglottic tumor extension, and thyroid cartilage periosteal or extralaryngeal invasion. Consequently, ESFR is generally contraindicated in T4 glottic laryngeal cancers exhibiting more than minimal thyroid cartilage invasion. The advantage of traditional CHEP lies in its broader applicability, whereas ESFR is most appropriately utilized in a carefully defined patient population.

The following considerations are crucial for both surgical techniques: 1. During reconstruction, maintain the anterior inclination of the arytenoid cartilage and ensure that sutures are appropriately tight to preserve its mobility. 2. When repairing the piriform fossa, employ moderate anterior displacement of the suture line to minimize postoperative swallowing errors. 2. Intraoperatively, take care to protect the recurrent laryngeal nerve to ensure recovery of piriform fossa function, the reflex mechanisms of the reconstructed larynx, and arytenoid cartilage mobility. At least one cricoarytenoid unit (including the cricoarytenoid muscle group) should be preserved to maintain vocal function in the reconstructed larynx. In ESFR, the central portion of the epiglottic valve is sutured to the thyroid cartilage to expand the pharyngeal cavity and prevent postoperative laryngeal stenosis.

Our findings indicate that ESFR outperforms traditional CHEP significantly in terms of postoperative extubation time and the incidence of swallowing errors at 8 weeks postoperatively. The significantly shorter extubation time observed in the ESFR group (8 vs. 18 days, *P*<0.01) likely stems from three interrelated mechanisms: anatomic preservation, reduced neurovascular trauma, and surgical technique-driven airway stability. In ESFR, the retained posterior thyroid cartilage offers structural support for the neoglottis, enabling earlier decannulation. The preserved suprahyoid muscles and intact piriform fossa anatomy decrease pharyngeal residue and aspiration rates. By maintaining the posterior thyroid lamina and cricoarytenoid units, ESFR prevents laryngeal collapse during swallowing. Avoiding cricothyroid joint dissection reduces superior laryngeal nerve injury, preserving laryngeal sensitivity. The epiglottic flap provides immediate glottic coverage, promoting faster mucosal healing than the exposed cricoid cartilage in CHEP.

We also assessed the severity of postoperative dysphagia and coughing in patients who underwent ESFR. The results revealed that the discomfort associated with these symptoms was significantly less pronounced in ESFR patients compared to those in the traditional CHEP group. This outcome aligns with our clinical expectations and facilitates improved patient recovery. Potential contributing factors include: 1. ESFR’s avoidance of upper and lower thyroid cartilage corner resection and cricothyroid joint dislocation, minimizing the risk of recurrent and superior laryngeal nerve injury; Furthermore, the surgical technique is more straightforward, and the intraoperative field of vision is improved. 2. ESFR’s preservation of the posterior inferior thyroid cartilage plate may elevate the larynx and trachea during swallowing, and maintain a more normal pyriform sinus configuration. Literature suggests pyriform sinus reduction is the only significant factor in aspiration reduction (14). Since the ESFR procedure maintains a relatively normal anatomical position, the reduction of the piriform fossa after surgery is faster than with traditional CHEP. Typically, after CHEP surgery, patients who accidentally swallow oral secretions are prone to aspiration. Reduced discomfort, such as choking and coughing, facilitates subsequent swallowing and drinking training, alleviates patient anxiety related to eating, and promotes the development of appropriate swallowing techniques. Earlier occlusion training and vocal exercises can then be implemented to restore laryngeal function (**Figure 1C, D**). Yücetürk et al. (13) believe that early extubation promotes timely restoration of phonation and swallowing, mitigates pulmonary infections resulting from aspiration of oral secretions, and consequently reduces both hospitalization duration and the risk of hospital-acquired infections. In summary, the advantages of ESFR include a reduction in the severity of postoperative dysphagia and coughing, leading to accelerated recovery of swallowing function and improved overall patient outcomes.

This study has several limitations. First, the retrospective design introduces potential selection bias. Second, the sample size remains modest, which may limit statistical power. Third, the single-center nature of this study warrants validation through multi-institutional trials. Additionly, the exclusion of tumors involving the preepiglottic space inherently restricts the comparability of ESFR and CHEP. The ESFR technique, although it offers potentially superior functional outcomes in select cases, is inherently limited by its reliance on a healthy epiglottic flap for successful reconstruction. A critical limitation of this approach is the exclusion of tumors involving the preepiglottic space. This restriction is grounded in sound oncological principles: infiltration of the preepiglottic space typically requires more extensive resection, which compromises the anatomical integrity needed to create a viable epiglottic flap for reconstruction. Thus, while ESFR may significantly enhance functionality for specific patient subsets, as demonstrated in the results, it is not intended to replace existing methodologies but rather to complement them. Finally, the retrospective design and its inherent constraints hinder our capacity to definitively assess the relative benefits and drawbacks of ESFR and CHEP. A prospective, randomized, controlled trial is needed to directly compare these techniques in a carefully selected patient population, stratified by T-stage (T2-T3) and excluding preepiglottic involvement, with standardized postoperative assessments. While this study provides preliminary data on the efficacy of ESFR, a prospective study is essential to definitively evaluate its clinical significance.

## 5 Conclusion

In conclusion, ESFR emerges as a promising alternative to CHEP for select laryngeal cancer patients, demonstrating superior early functional recovery, as indicated by reduced dysphagia and accelerated extubation, while maintaining comparable long-term survival rates. The success of ESFR is largely attributed to its preservation of key laryngeal structures and reduced risk of nerve damage. However, its application is limited to cases without preepiglottic space involvement, underscoring the importance of stringent patient selection. Although CHEP remains a viable option for more advanced cases, ESFR offers a distinct advantage when anatomical criteria are met. To fully elucidate the clinical significance of ESFR and optimize patient selection, future prospective, randomized trials are essential.

## Data Availability

The datasets presented in this article are not readily available because our raw data are all state secret level data, and papers are reviewed to see if they pose a risk of leakage. Requests to access the datasets should be directed to 15680483625@163.com.
